# Hu-Lu-Ba-Wan Attenuates Diabetic Nephropathy in Type 2 Diabetic Rats through PKC-****α****/NADPH Oxidase Signaling Pathway

**DOI:** 10.1155/2013/504642

**Published:** 2013-06-25

**Authors:** Lishan Zhou, Hui Dong, Yi Huang, Lijun Xu, Xin Zou, Kaifu Wang, Guang Chen, Fuer Lu

**Affiliations:** ^1^Institute of Integrated Traditional Chinese and Western Medicine, Tongji Hospital, Tongji Medical College, Huazhong University of Science and Technology, Wuhan, Hubei 430030, China; ^2^Department of Integrated Traditional Chinese and Western Medicine, Wuhan Children's Hospital, Wuhan, Hubei 430016, China; ^3^Department of Nephrology, Tongji Hospital, Tongji Medical College, Huazhong University of Science and Technology, Wuhan, Hubei 430030, China; ^4^Department of Integrated Traditional Chinese and Western Medicine, Tongji Hospital, Tongji Medical College, Huazhong University of Science and Technology, Wuhan, Hubei 430030, China

## Abstract

Hu-Lu-Ba-Wan (HLBW) is a Chinese herbal prescription used to treat kidney deficiency. The aim of this study was to explore the effect and mechanism of HLBW on diabetic nephropathy (DN) in type 2 diabetic rats. The rat model of DN was established by being fed a high-fat diet and intravenous injection of streptozotocin. Then, HLBW decoction was administered for 16 weeks. Blood glucose level, lipid profile, renal function, 24-hour total urinary protein, and albumin content were examined. Renal morphology and superoxide anion levels were evaluated. The activity of nicotinamide-adenine dinucleotide phosphate (NADPH) and protein kinase C-alpha (PKC-**α**) related genes expression in renal tissue were also determined. Our data demonstrated that HLBW significantly improved hyperglycemia, hyperlipidemia, and proteinuria in diabetic rats compared with those of control group. HLBW also alleviated glomerular expansion and fibrosis, extracellular matrix accumulation and effacement of the foot processes. Additionally, HLBW reduced superoxide anion level, NADPH oxidase activity, the protein and mRNA expressions of p47^phox^, and the protein expression of phosphorylated PKC-**α** in renal tissue. These results suggest that HLBW is effective in the treatment of DN in rats. The underlying mechanism may be related to the attenuation of renal oxidative stress via PKC-**α**/NADPH oxidase signaling pathway.

## 1. Introduction

The epidemic of diabetes mellitus (DM) is rapidly becoming a severe public health problem, especially in China. It is reported that the prevalence of diabetes has reached 9.7%, accounting for 92.4 million adults with diabetes [1]. The morbidity and mortality associated with this disease derives primarily from its microvascular and macrovascular complications caused by persistent hyperglycemia [2]. Diabetic nephropathy (DN), with hidden symptoms in early stage and lack of effective therapy, is the most common microvascular complication of diabetes [3]. Accumulating evidence has demonstrated that hyperglycemia and the resulting oxidative stress play important roles in the development of DN [4–6]. Therefore, therapies designed to improve hyperglycemia and reduce oxidase stress may be promising in the treatment of DN.

Plant-based medicines are widely used to treat diabetes and its complications in the local clinics of China. In Chinese medicine, DN is referred to kidney deficiency disease. Therefore, prescriptions reinforcing kidney may have the potential to treat DN. Among effective prescriptions, Hu-Lu-Ba-Wan (HLBW), which consisted of *Trigonella foenum-graecum* L. (TFG) and *Psoralea corylifolia* L. (PC), attracts much attention for their novel kidney reinforcing efficacy. Many researches have shown the beneficial effect of TFG and PC on improving hyperglycemia and alleviating inflammation [7–13]. In a recent, their antioxidative activity and renal protective effect have also been documented [14–19]. However, these individual studies just included incomplete description in this aspect. The effect of HLBW, which is the combination of TFG and PC, on DN is rarely reported. Therefore, the aim of this study was to explore the effect of HLBW on DN in type 2 diabetic rats and investigate its possible oxidative stress against mechanism.

## 2. Materials and Methods

### 2.1. Animals

Male Wistar rats weighting 180~200 g, obtained from Hubei province Center for Disease Control and Prevention, were used in this study. The rats were maintained at ambient temperature (22°C ± 1°C) with a 12 : 12 h light-dark cycle and free access to water and food. The research was conducted in accordance with the internationally accepted principles for laboratory animal use and care as found in the Chinese guidelines.

### 2.2. Preparation of HLBW

TFG and PC were purchased from Traditional Chinese Medicine Company in Hubei Province (Wuhan, China) and authenticated by the Department of Pharmacognscy, Hubei University of Chinese Medicine (Wuhan, China). The rat doses of TFG and PC were obtained by the conversion of human doses (Chinese Pharmacopeia, 2010) to rat equivalent doses based on body surface areas. The weight ratio of TFG to PC is 1 : 1. HLBW preparation process was as follows. The dry seeds were crushed into powder and soaked overnight before boiled. After cooling down, the decoction was deposited in 95% ethanol (1 : 1 v/v) overnight at room temperature. The precipitate from the decoction was separated by a filter. Then, the alcoholic filtrate was distilled to remove the ethanol and was concentrated by Rotavapor (BUCHI, Flawil, Switzerland). The final liquid extracted from 1 g HLBW (which contains 0.5 g TFG and 0.5 g PC), 1 g TFG, and 1 g PC was 1.32 mL, 0.63 mL, and 0.66 mL, respectively.

### 2.3. Animal Modeling, Grouping, and Treatment

After adaptive feeding with standard rat diet (containing 35% flour, 20% soy meal, 20% corn meal, 15.5% bran, 0.5% bean oil, 5% fish meal, 2.5% bone meal, 1% dusty yeast, and 0.5% salt) for one week, eight rats were selected randomly as the normal control group (control). The rats in the control group continued their standard diet, while the remaining rats were fed with a high-fat diet (containing 67.5% standard laboratory rat chow, 15% lard, 15% sugar, 2% cholesterol, and 0.5% bile salts) for 4 weeks. Then, they were injected with streptozotocin (STZ, Sigma Chemical Co. MO, USA, 30 mg/kg) into the tail vein after an overnight fast. One week later, oral glucose tolerance test (OGTT) was performed. The 95% range of confidence was calculated according to the plasma glucose levels of normal rats. Rats with impaired glucose tolerance (IGT) (Plasma glucose levels of rats at two time points were higher than the upper limit at two time points, or 20% higher than the upper limit at one time point.) were selected. Then the IGT rats were randomized into the untreated diabetic control group (Diabetic), TFG treated group (TFG), PC treated group (PC), HLBW treated group (HLBW), and Captopril (Sino-American Shanghai Squibb Pharmaceuticals Ltd., Shanghai, China) treated group (Captopril). Rats in the previous mentioned treatment groups were administered with corresponding therapy, TFG (9 g/kg/d), PC (9 g/kg/d), HLBW (18 g/kg/d), or Captopril (10 mg/kg/d), intragastrically for 16 weeks. Oral gavage was performed once a day between 8:00 and 10:00 a.m. Rats in untreated diabetic and normal control group were administered with the same volume of distilled water. Doses were adjusted to the body weight recorded once a week.

### 2.4. Sampling

All the animals were sacrificed at the end of the 16th week. Urine samples of 24 hours were collected by metabolic cages the day before sacrifice. Blood samples were obtained by aorta abdominalis puncture at the time of sacrifice. After centrifuging at 3000 r/min for 30 min at 4°C, the specimen serum was collected and stored at −20°C until analysis. Meanwhile, kidneys were quickly taken out by laparotomy and flushed with normal saline on ice. Then, the entire kidney mass was scaled. Part of the left renal samples was, respectively, fixed in 4% paraformaldehyde solution for paraffin embedding or fixed in 2.5% glutaraldehyde solution for transmission electron microscope (TEM), while the other part was prepared for frozen sections. The right renal samples were preserved at −80°C until use. 

### 2.5. OGTT and Biochemical Analysis

After the rats fasted for 12 hours, 50% glucose at a dose of 2 g/kg was orally administered. Then, blood samples were collected from tail veins at 0 (prior to glucose loading), 60 and 120 minutes (after glucose loading). Blood glucose level was examined by glucose-oxidase method using a glucose monitor (LifeScan Inc., J&J Company, Milpitas, CA, USA). Serum levels of total cholesterol (TC), triglycerides (TG), low-density lipoprotein cholesterol (LDL-C), high-density lipoprotein cholesterol (HDL-C), blood urea nitrogen (BUN), and serum creatinine (SCr) were determined using commercial reagents (Jiancheng Bioengineering Institute, Nanjing, China). Urinary total protein and albumin concentrations were measured by the immunoturbidimetric method using a biochemical analyzer (Roche, Basel, Switzerland).

### 2.6. Renal Histological Studies

The paraffin slides were stained with hematoxylin and eosin (HE) to evaluate the histology of glomerulus, periodic acid-schiff (PAS) to evaluate the thickening of glomerular basement membrane and hyperplasia of mesangium, and Masson's trichrome stain (Masson) to evaluate the fibrosis of glomerulus, which were all observed under optical microscope. The following criteria were used for scoring renal histology. A semiquantitative score (sclerosis index (SI)) was used to evaluated the degree of glomerulosclerosis [20]. Severity of sclerosis for each glomerulus was graded from 0 to 4+ as follows: 0 represents no lesion, 1+ represents sclerosis of <25% of the glomerulus, while 2+, 3+, and 4+ represent sclerosis of 25% to 50%, >50% to 75%, and >75% of the glomerulus. The whole kidney average sclerosis index on one section was obtained by averaging scores from all glomeruli. Renal ultrastructure was also observed under a transmission electron microscope (FEI Tecnai G^2^12, Holland).

### 2.7. Detection of Renal Superoxide Anion Levels

Dihydroethidium (DHE), an oxidant-sensitive probe, is widely used for detection of reactive oxygen species (ROS). Two products of DHE oxidation, ethidium and 2-hydroxyethidium, can bind to the nuclear DNA, thereby forming a strong red fluorescent complex [21]. Frozen sections of the kidney (8 *μ*m) were placed on glass slides and incubated with DHE (10 mmol/L, Beyotime Institute of Biotechnology, Shanghai, China) in a dark container at 37°C for 30 min. After rinsing in PBS three times, the sections were viewed with an inverted microscope (Nikon, Tokyo, Japan) [22].

### 2.8. Measurement of Renal NADPH Activity

Renocortical tissues were homogenated and lyzed in mammal tissue protein extraction reagent. Then, the extracted protein was supplemented with protease inhibitor cocktail and phenylmethylsulfonylfluoride (PMSF) (Guge shengwu Technology Co., Wuhan, China). After centrifuged at 12000 r/min for 30 min at 4°C, the supernatant was collected to quantify the protein concentration with BCA protein assay kit (Beyotime Institute of Biotechnology, Shanghai, China). Renal NADPH activity was measured using an NADPH Activity Quantification Kit (Genmed Scientifics Inc., Shanghai, China).

### 2.9. Western Blot Analysis

Renocortical extracts (100 *μ*g protein) were mixed with sample buffer, boiled for 10 min, and subjected to 10% SDS-PAGE gel (100 v, 2 h). Separated proteins on the gel were transferred to nitrocellulose membranes. The membranes were then blocked with 5% fat-free dry milk in TBST or 0.5% bovine serum albumin (BSA) for 2 h at room temperature, followed by overnight incubation at 4°C with antibodies (p47^phox^, phosphorylated PKC-*α*, fibronectin, and *β*-actin) (Abcam, Hong kong, China). After washed by TBST three times, the membranes were lucifugally incubated with the dylight 800-labeled antibody to rabbit IgG (H+L) (KPL Company, Hongkong, China) at room temperature for 1 h. Then, the membranes were lucifugally washed with TBST three times. Immunoreactive proteins were detected by near infrared double color laser imaging system (Odyssey, Lincoln, USA). Band densities were determined by Bio-Rad Quantity One software and quantified as the ratio between OD value of target band to OD value of *β*-actin.

### 2.10. Quantitative RT-PCR Analysis

Total RNA was extracted from the renocortical tissue with Trizol reagent according to the manufacturer's instructions. RNA purity and concentration were measured by a Nucleic Acid/Protein Analyzer (Thermo, Rockford, USA). The extracted total RNA (1 *μ*g) was reverse transcribed with PrimeScript RT reagent Kit (TaKaRa Company, Dalian, China) on a Mastercycler gradient PCR apparatus (Eppendorf Company, Hamburg, Germany). The cDNA was kept at −20°C prior to PCR amplification. Real-time PCR reactions were performed in 48-well optical PCR plates using SYBR Premix Ex Taq (TaKaRa Company, Dalian, China) on an Applied Biosystems StepOne Real-Time PCR System (Stepone, Foster City, USA). A 2^−ΔΔCT^ was used for analyzing the data. Primer sequences are listed in Table 1.

### 2.11. Statistical Analysis

All data are presented as mean ± standard deviation (SD) and analyzed by SPSS19.0 Statistical Software. Statistical significance was determined by one-way analysis of variance (ANOVA). Data with equal variances were not assumed followed by Dunnett's T3 test, while data with equal variances assumed followed by LSD test. A probability of less than 0.05 was considered to be statistically significant.

## 3. Results

### 3.1. HLBW Improved the Glucose Tolerance of Rats with DN

Rats with untreated DN showed severe hyperglycemia characterized by elevated fasting and postprandial plasma glucose levels (*P* < 0.01). However, treatment with HLBW and its single components, as well as Captopril, significantly decreased plasma glucose levels compared with those of untreated diabetic rats (*P* < 0.01) (Table 2).

### 3.2. HLBW Improved Plasma Lipid Profiles of Rats with DN

 As shown in Table 3, rats with untreated DN showed severe dyslipidemia. The serum TC, TG, and LDL-C levels increased compared with those of control rats (*P* < 0.01). Treatment with HLBW and its single components markedly alleviated hyperlipidemia in rats with DN (*P* < 0.01, *P* < 0.05, resp.). However, Captopril did not show any beneficial effect on dyslipidemia when compared with diabetic rats.

### 3.3. HLBW Improved Renal Function and Proteinuria of Rats with DN

 As shown in Table 4, rats with untreated DN exhibited an elevation in the term of ratio of kidney to body weight, as well as severe renal dysfunction and proteinuria. The BUN, SCr, urinary total protein, and albumin concentrations increased significantly in comparison to those of control rats (*P* < 0.01). However, treatment with HLBW and its single components, as well as Captopril, significantly reduced the ratio of kidney to body weight and reversed renal dysfunction and proteinuria (*P* < 0.01).

### 3.4. HLBW Improved Renal Morphology Changes of Rats with DN

**Figure 1:**
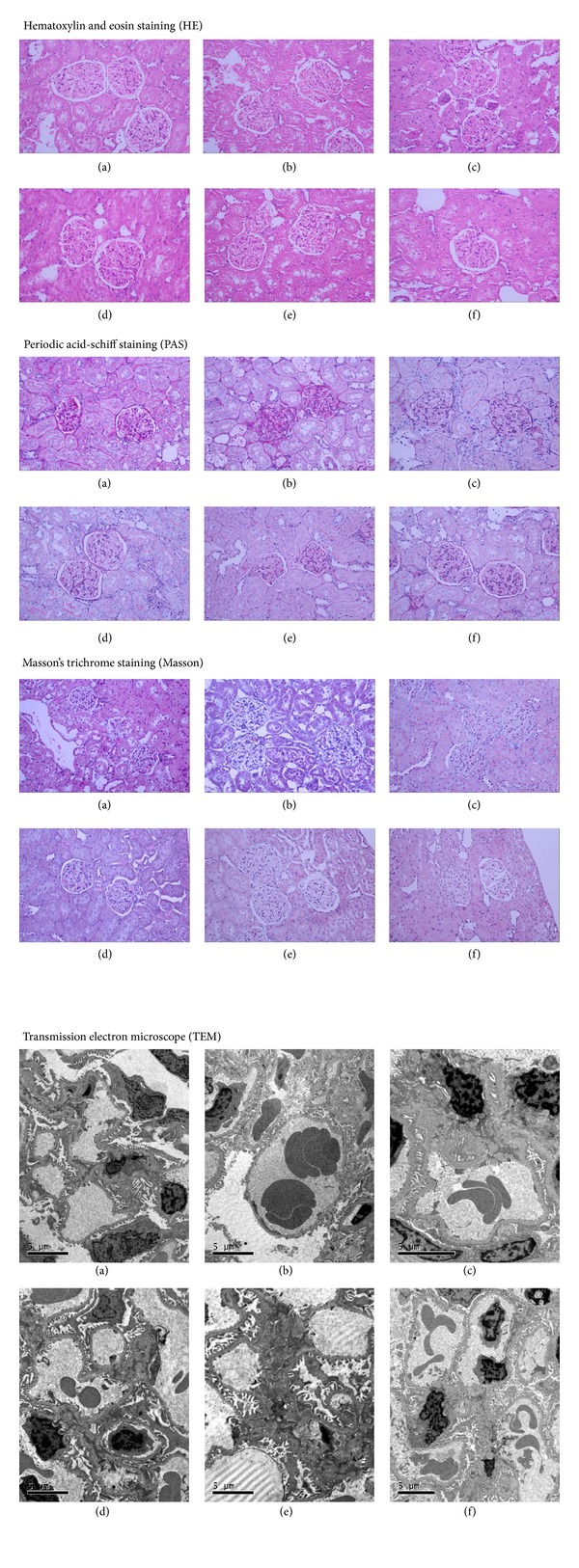
Morphological pictures of the glomerulus. (a) Control group; (b) Diabetic group; (c) TFG group; (d) PC group; (e) HLBW group; (f) Captopril group.

**Figure 2:**
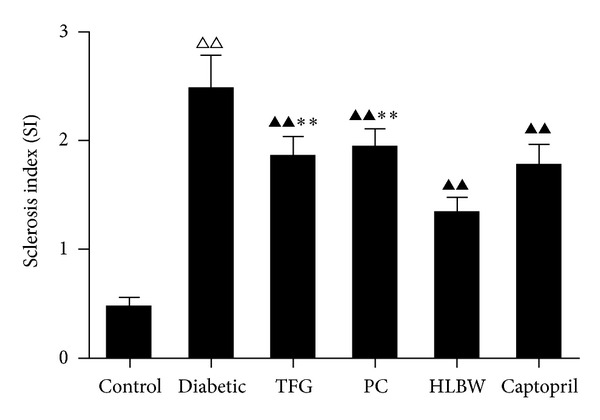
The effect of HLBW on glomerular sclerosis index (SI) of rats with DN. Values are mean ± SD (*n* = 5). ^△△^ *P* < 0.01 versus the control group, ^▲▲^ *P* < 0.01 versus the untreated diabetic group, and **P < 0.01 versus HLBW group.

As shown in Figure 1, renal tissues of rats with untreated DN showed remarkable glomerular hypertrophy and fibrosis, hyperplasia of mesangial area, and effacement of the podocyte foot processes. As shown in Figure 2, SI of rats with untreated DN elevated significantly. However, treatment with HLBW restored these morphology changes and SI level. Treatment with either TFG or PC also attenuated glomerular hypertrophy and fibrosis, mesangial hyperplasia, podocyte foot processes effacement, and glomerular SI level.

### 3.5. HLBW Decreased Renal Superoxide Anion Production of Rats with DN

**Figure 3:**
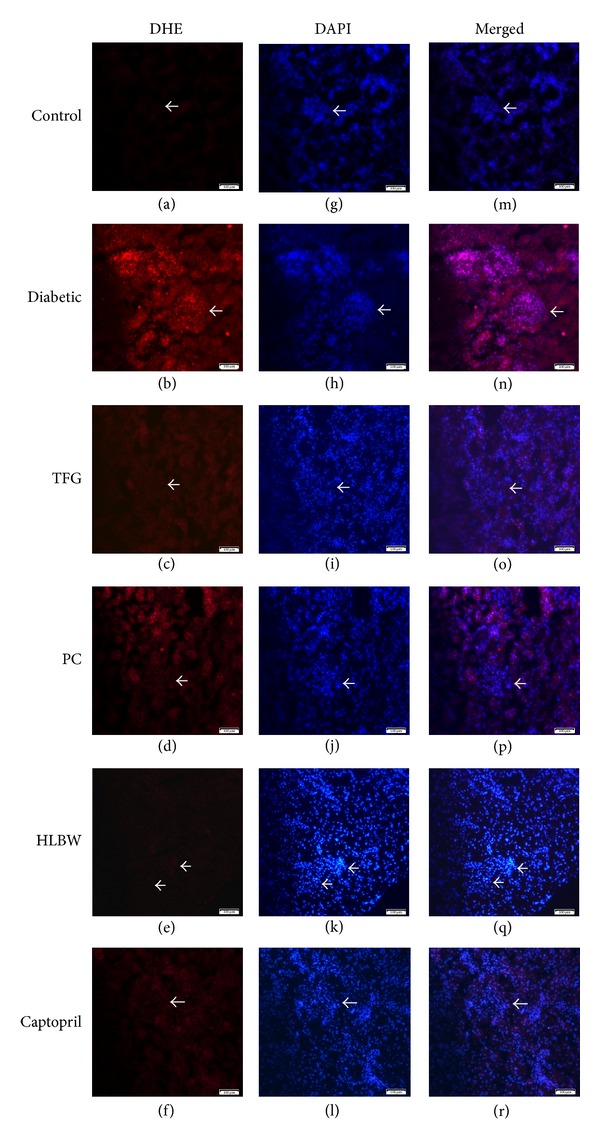
Dihydroethidium staining in the glomeruli from the rats in different groups. (original magnification: ×100). (a)–(f) Visualization of ROS in the glomeruli using DHE stains. (g)–(l) Visualization of nucleus in the glomeruli using DAPI stains. (m)–(r) The superimposed pictures of different groups. (Arrows are pointing at the glomerulus.)

As shown in Figure 3, a high level of DHE fluorescence, indicating the increased superoxide anion production [23, 24], was observed in renal tissues of DN rats. However, treatment with HLBW and its single components, as well as Captopril, significantly reduced the level of DHE fluorescence in renal tissues of rats with DN.

### 3.6. HLBW Decreased Renal NADPH Activity of Rats with DN

**Figure 4:**
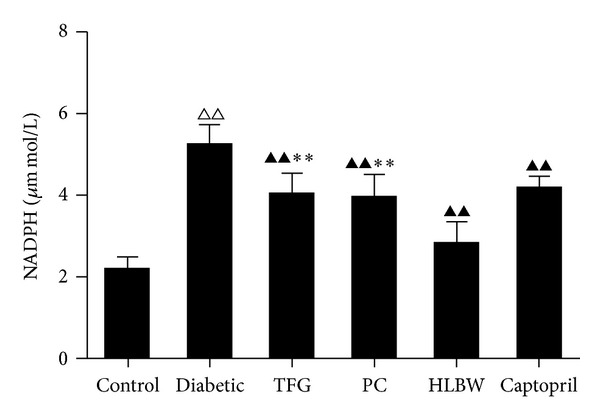
The effect of HLBW on renal NADPH activity of rats with DN. Values are mean ± SD (*n* = 8). ^△△^ *P* < 0.01 versus the control group, ^▲▲^ *P* < 0.01 versus the untreated diabetic group, and **P < 0.01 versus HLBW group.

As shown in Figure 4, the activity of renal NADPH was much higher in DN rats than that in control rats (*P* < 0.01). After the treatment with HLBW, TFG, PC, or Captopril, the activity of renal NADPH was significantly decreased in rats with DN (*P* < 0.01). Furthermore, a significant difference in renal NADPH activity was identified between HLBW and its single components, indicating a better renal NADPH decreasing activity of HLBW than that of TFG and PC (*P* < 0.01).

### 3.7. HLBW Decreased Renal PKC-*α*, Phosphorylated PKC-*α*, p47^phox^, and Fibronectin Gene Expressions of Rats with DN

**Figure 5:**
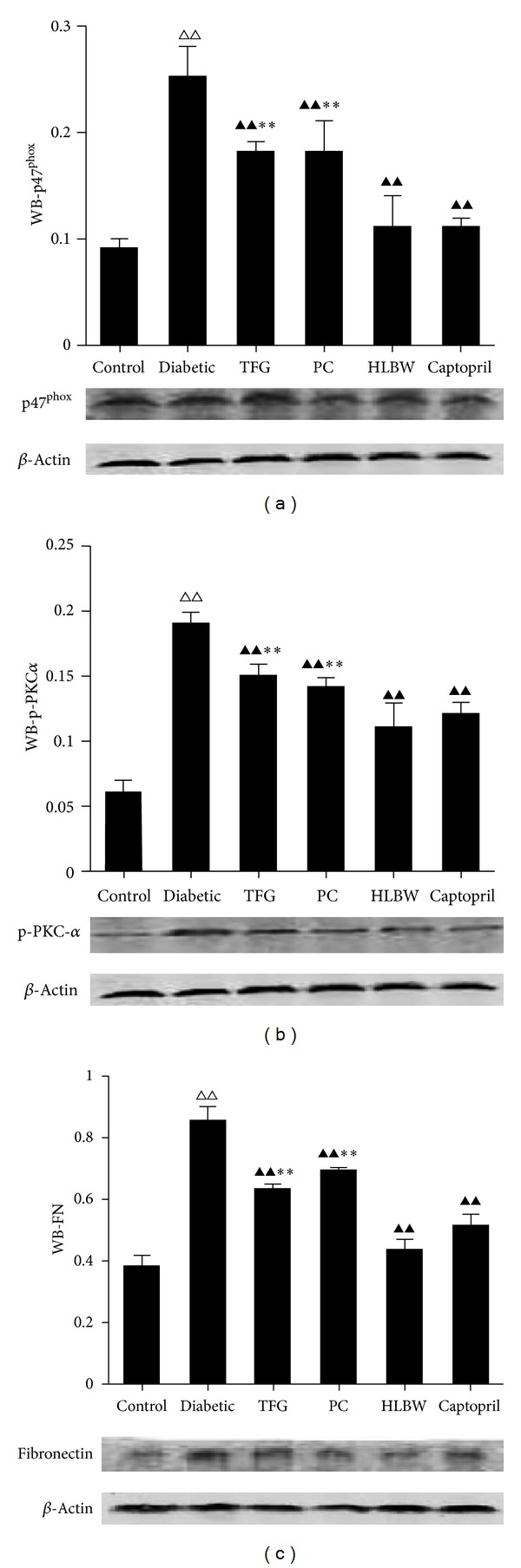
The effect of HLBW on the expression of renal proteins: (a) p47^phox^; (b) p-PKC-*α*; (c) fibronectin. Values are mean ± SD (*n* = 8). ^△△^ *P* < 0.01 versus the control group, ^▲▲^ *P* < 0.01 versus the untreated diabetic group, and **P < 0.01 versus HLBW group.

**Figure 6:**
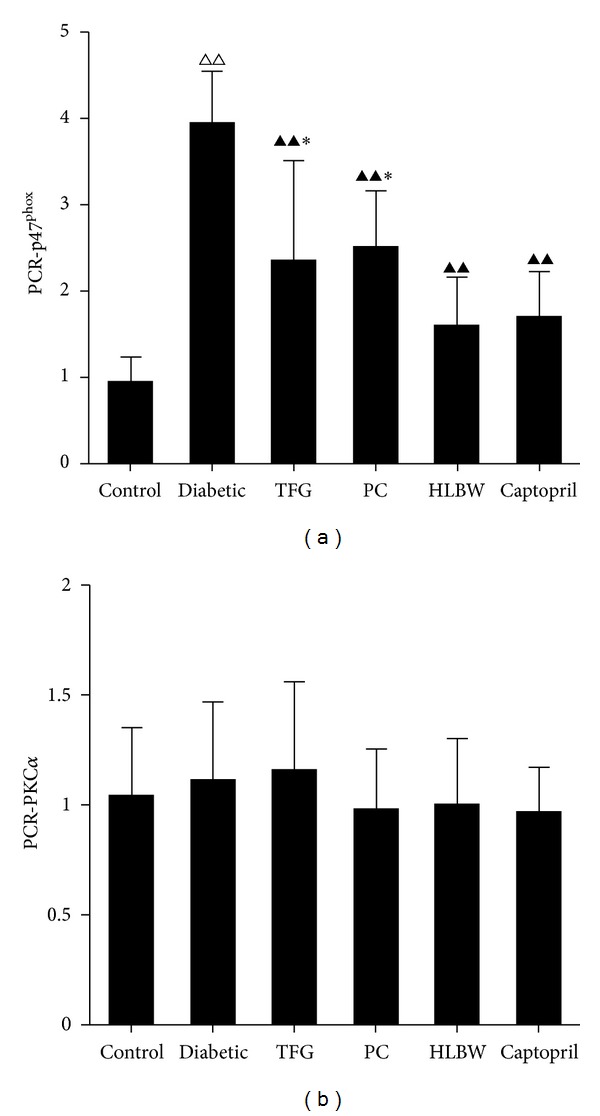
The effect of HLBW on the expression of renal p47^phox^ (a) and PKC-*α* (b) mRNA. Values are mean ± SD (*n* = 8). ^△△^ *P* < 0.01 versus the control group, ^▲▲^ *P* < 0.01 versus the untreated diabetic group, and *P < 0.05 versus HLBW group.

As shown in Figures 5 and 6, renal p47^phox^ protein level and mRNA concentration significantly increased in DN rats compared with those in control rats (*P* < 0.01). However, there was a significant reduction in the expression of p47^phox^ protein and mRNA in all the treatment groups (*P* < 0.01). Moreover, a marked reduction in the expression of p47^phox^ protein and mRNA was identified after HLBW treatment in contrast to TFG or PC treatment alone (*P* < 0.01, *P* < 0.05). 

As shown in Figures 5 and 6, renal phosphorylated PKC-*α* protein level increased significantly in rats with untreated DN (*P* < 0.01). HLBW, TFG, PC and Captopril treatment significantly reduced the expression of phosphorylated PKC-*α* protein (*P* < 0.01). Additionally, HLBW, better than TFG or PC alone, significantly decreased phosphorylated PKC-*α* protein expression (*P* < 0.01). However, in terms of PKC-*α* mRNA expression, no significant difference was identified between the groups.

With regard to Fibronectin, which is a marker of renal fibrosis, rats with untreated DN exhibited a significant increase of renal Fibronectin protein expression (*P* < 0.01). HLBW, treatment with TFG, PC, and Captopril significantly reduced the expression of renal fibronectin protein (*P* < 0.01). HLBW also better than TFG or PC alone, significantly decreased renal expression of fibronectin protein (*P* < 0.01).

## 4. Discussion

HLBW is a Chinese herbal prescription that is just composed of two herbs, TFG and PC. Previous evidence from animal and clinical studies has suggested that TFG has the potential to treat hyperglycemia, hyperlipidemia, and renal disease [7–10, 18]. However, the comparisons between the effect of HLBW and its single components on DN have rarely been reported.

In our study, the rat model of type 2 diabetes was successfully established by a high-fat diet accompanied by intravenous injection of relatively small doses of STZ. The animals manifested the characteristics of hyperglycemia and hyperlipidemia. BUN, SCr, 24-hour urinary total protein, and albumin concentrations significantly increased at the same time, which indicated the development of DN. HLBW showed hypoglycemia, hypolipidemia, and renal protection against DN. Meanwhile, such the beneficial outcomes were also confirmed in DN rats treated with TFG or PC alone. With regard to renal morphology changes, the untreated diabetic rats were characteristized of remarkable glomerular expansion and fibrosis, extracellular matrix (ECM) accumulation, and effacement of the foot processes. HLBW, also similar to TFG and PC, attenuated these histopathological abnormalities in renal tissue in diabetic rats. However, HLBW, better than TFG and PC, reduced the expression of renal fibronectin protein and the level of SI. Since fibronectin is a predominant matrix protein representing the degree of renal fibrosis and SI represents the extent of glomerular sclerosis, this result indicates that HLBW may be promising at ameliorating renal fibrosis.

In diabetic patients, hyperglycemia and hyperlipidemia enhance the oxidative stress which is involved in the mechanism of diabetic vasculopathy. The oxidative stress can also aggravate the glucolipid metabolism disorder, thereby forming a vicious circle [5, 6, 25]. Excessive reactive oxygen species (ROS) generated by oxidative stress not only induces oxidative damage via peroxidation of the biomacromolecule but also interferes with cell signal transduction via serving as a second messenger [26]. To evaluate superoxide production in the kidneys, dihydroethidium (DHE) staining is often performed. In the presence of superoxide, DHE is changed into ethidium bromide, which binds to DNA and exhibits red fluorescence in the nucleus [27–29]. In our study, a massive increase in superoxide anion generation was identified in the diabetic renal tissues. It was attenuated by HLBW and its single components. However, HLBW treatment, compared with TFG or PC, showed the maximum decreasing of superoxide production, indicating the efficient oxidative stress protection in DN.

In order to elucidate the protection mechanisms of HLBW against oxidative stress in renal tissues, we further evaluated the gene expressions involved in the production of ROS. Among the multiple sources in the diabetic kidney, ROS derived from NADPH oxidase is crucial to the development of DN [30, 31]. Accordingly, we further examined the activity of NADPH oxidase and the gene expressions of its upstream regulator, protein kinase C-*α* (PKC-*α*), and p47^phox^, which are the regulatory subunit of NADPH oxidase [26]. The results showed that HLBW significantly inhibited NADPH oxidase activity and p47^phox^ gene expression in renal tissues of diabetic rats. HLBW also decreased phosphorylated PKC-*α* protein expression. These effects were also superior to that observed in DN rats treated with TFG or PC alone. However, no significant difference was identified between the groups regarding the expression of PKC-*α* mRNA. It might give a hint that the effect of HLBW on PKC-*α* is posttranscription. Therefore, our study firstly demonstrated the inhibitory effect of HLBW on PKC-*α*/NADPH oxidase signaling pathway, which may attribute to the reduction of ROS production in renal tissues.

In summary, our study demonstrates that HLBW and its single components, TFG and PC, improve renal function and ameliorate renal histopathological alterations in type 2 diabetic rats. The mechanism may be related to reducing oxidative stress via PKC-*α*/NADPH oxidase signaling pathway. Moreover, HLBW exhibits a better efficacy than TFG or PC alone on protecting against oxidative stress in DN, which indicates a theory of prescription compatibility in traditional Chinese medicine.

## Figures and Tables

**Table 1:** Real-time PCR primer sequences.

Gene	Forward (5′ → 3′)	Reverse (5′ → 3′)
*β*-Actin	5′-GGAGATTACTGCCCTGGCTCCTA-3′	5′-GACTCATCGTACTCCTGCTTGCTG-3′
p47^phox^	5′-GGACACCTTCATTCGCCACA-3′	5′-GTCCTGCCACTTAACCAGGAACA-3′
PKC-*α*	5′-TCCAGGATGACGACGTGGAG-3′	5′-CGTTGACGTATTCCATGACGAAG-3′

**Table 2:** The effect of HLBW on OGTT in rats with DN.

Group	FBG (mmol/L)	PG-1 h (mmol/L)	PG-2 h (mmol/L)
Control	5.84 ± 0.55	6.15 ± 0.37	6.05 ± 0.60
Diabetic	15.81 ± 1.99^△△^	27.10 ± 4.52^△△^	23.29 ± 3.77^△△^
TFG	5.76 ± 0.44^▲▲^	10.11 ± 2.97^▲▲^	6.51 ± 1.32^▲▲^
PC	5.75 ± 1.30^▲▲^	9.81 ± 3.71^▲▲^	6.26 ± 1.13^▲▲^
HLBW	5.73 ± 1.01^▲▲^	8.08 ± 0.91^▲▲^	6.01 ± 0.91^▲▲^
Captopril	8.50 ± 1.58^▲▲^	14.90 ± 5.78^▲▲^	9.84 ± 1.64^▲▲^

Values are mean ± SD (*n* = 8). ^△△^ *P* < 0.01 versus the control group, ^▲▲^ *P* < 0.01 versus the untreated diabetic group. FBG: fasting blood glucose; PG-1 h: postprandial blood glucose at 1 hour after glucose loading; PG-2 h: postprandial blood glucose at 2 hours after glucose loading.

**Table 3:** The effect of HLBW on plasma lipid profiles in rats with DN.

Group	TG (mmol/L)	TC (mmol/L)	LDL-C (mmol/L)	HDL-C (mmol/L)
Control	1.04 ± 0.16	2.09 ± 0.29	0.51 ± 0.15	1.50 ± 0.24
Diabetic	3.48 ± 1.14^△△^	7.91 ± 1.53^△△^	4.46 ± 1.57^△△^	1.13 ± 0.18
TFG	1.23 ± 0.38^▲▲^	4.73 ± 0.74^▲▲^	1.29 ± 0.36^▲▲^	1.40 ± 0.36
PC	1.49 ± 0.19^▲^	5.06 ± 1.05^▲^	1.71 ± 0.51^▲^	1.35 ± 0.35
HLBW	1.18 ± 0.23^▲▲^	4.33 ± 0.59^▲▲^	1.20 ± 0.28^▲▲^	1.46 ± 0.29
Captopril	1.96 ± 0.80	7.23 ± 1.62	2.84 ± 0.76	1.28 ± 0.16

Values are mean ± SD (*N* = 8). ^△△^ *P* < 0.01 versus the control group, ^▲^ *P* < 0.05, ^▲▲^ *P* < 0.01 versus the untreated diabetic group. TG: triglyceride; TC: total cholesterol; LDL-C: low-density lipoprotein cholesterol; HDL-C: high-density lipoprotein cholesterol.

**Table 4:** The effect of HLBW on renal function and proteinuria of rats with DN.

Group	Kidney/body weight (%)	BUN (mmol/L)	SCr (*μ*mol/L)	Urinary total protein (*μ*g/24 h)	Urinary albumin (*μ*g/24 h)
Control	0.42 ± 0.04	3.96 ± 0.81	146.86 ± 12.39	6.71 ± 2.39	0.08 ± 0.03
Diabetic	0.64 ± 0.12^△△^	11.46 ± 1.50^△△^	229.61 ± 30.10^△△^	50.79 ± 7.41^△△^	3.54 ± 1.19^△△^
TFG	0.48 ± 0.08^▲▲^	5.68 ± 1.53^▲▲^	184.89 ± 15.72^▲▲^	13.40 ± 5.22^▲▲^	0.41 ± 0.35^▲▲^
PC	0.43 ± 0.06^▲▲^	5.70 ± 1.02^▲▲^	183.34 ± 23.61^▲▲^	17.30 ± 8.30^▲▲^	0.54 ± 0.37^▲▲^
HLBW	0.42 ± 0.04^▲▲^	4.88 ± 0.93^▲▲^	165.16 ± 16.56^▲▲^	10.74 ± 6.62^▲▲^	0.31 ± 0.21^▲▲^
Captopril	0.45 ± 0.06^▲▲^	6.69 ± 1.78^▲▲^	190.13 ± 21.62^▲▲^	14.00 ± 4.08^▲▲^	0.40 ± 0.28^▲▲^

Values are mean ± SD (*n* = 8). ^△△^ *P* < 0.01 versus the control group, ^▲▲^ *P* < 0.01 versus the untreated diabetic group. BUN: blood urea nitrogen; SCr: serum creatinine.
